# High prevalence of tetracycline resistance in *Neisseria gonorrhoeae* across 22 European countries, 2024

**DOI:** 10.2807/1560-7917.ES.2026.31.2.2500965

**Published:** 2026-01-15

**Authors:** Susanne Jacobsson, Michelle J Cole, Melissa Jansen van Rensburg, Daniel Schröder, Otilia Mårdh, Csaba Ködmön, Magnus Unemo, the Euro-GASP tetracycline study group, Collaborators: Euro-GASP tetracycline study group, Sonja Pleininger, Stefanie Schindler, Ziad El-Khatib, Irith De Baetselier, Dominique Van Beckhoven, Dorien Van den Bossche, Amaryl Lecompte, Ivva Philipova, Panayiota Maikanti Charalambous, Despo Pieridou, Olga Apostolou, Hana Zákoucká, Helena Žemličková, Vladislav Jakubů, Steen Hoffmann, Maria Wessman, Beatrice Bercot, Emilie Chazelle, Cheick Kounta, Gilles Delmas, Dagmar Heuer, Regina Selb, Klaus Jansen, Vivi Miriagou, Eirini Siatravani, Dimitra Paraskeva, Eszter Balla, Lena Rós Ásmundsdóttir, Anna Margret Gudmundsdottir, Marianna Thordardottir, Kristjan Orri Helgason, Brendan Crowley, Sinead Saab, Derval Igoe, Mark Campbell, Angeline McIntyre, Paola Stefanelli, Barbara Suligoi, Monique Perrin, Francesca Mifsud, Julie Haider, Robert Cassar, Bente Børud, Hilde Kløvstad, Beata Młynarczyk-Bonikowska, Maria José Borrego, Peter Pavlik, Alexandra Bražinová, Julija Germ, Tanja Kustec, Raquel Abad, Julio Vazquez Moreno, Javier Gómez Castellá

**Affiliations:** 1 WHO Collaborating Centre for Gonorrhoea and Other STIs, Örebro University, Örebro, Sweden; 2 UK Health Security Agency (UKHSA), London, United Kingdom; 3 European Centre for Disease Prevention and Control (ECDC), Stockholm, Sweden; 4 Institute for Global Health, University College London (UCL), London, United Kingdom; 5 Euro-GASP study group members are listed under Collaborators and at the end of the article

**Keywords:** *Neisseria gonorrhoeae*, gonorrhoea, tetracycline, doxycycline, doxycycline post-exposure prophylaxis (doxy-PEP), prevention

## Abstract

In 2024, based on the European Committee on Antimicrobial Susceptibility Testing breakpoint, we observed a tetracycline resistance prevalence of 62.3% (2,231/3,579) in *Neisseria gonorrhoeae* isolates from 22 European countries (range: 16.5–100%). Multivariable analysis of correlations between resistance and patients’ epidemiological characteristics found tetracycline resistance associated with men who have sex with men (aOR: 1.38; 95% CI: 1.06–1.79). Our results are important when considering measures against transmission of sexually transmitted bacterial infections in Europe, such as in the context of doxycycline post-exposure prophylaxis (doxy-PEP).

Doxycycline post-exposure prophylaxis (doxy-PEP), administered as 200 mg within 24–72 hours after condomless sex to men who have sex with men (MSM) and transgender women with a bacterial sexually transmitted infection (STI) during the last year, has been shown in randomised controlled clinical studies and real-world settings to significantly reduce incident syphilis and chlamydia cases, but with more limited or no impact on gonorrhoea due to tetracycline resistance [1-6]. To further inform public health policy decision in relation to doxy-PEP, we present the latest results of tetracycline susceptibility testing in *Neisseria gonorrhoeae* isolates from 22 European Union/European Economic Area (EU/EEA) countries in 2024, generated through the European Gonococcal Antimicrobial Surveillance Programme (Euro-GASP) [7], with analysis by time (2022–2024) and demographic or epidemiological characteristics.

## *Neisseria gonorrhoeae* tetracycline susceptibility and resistance, 2024

In 2024, isolates of *N. gonorrhoeae* (n = 3,579) were obtained from 22 EU/EEA countries in Euro-GASP. Details regarding Euro-GASP structure, sampling, culture, antimicrobial susceptibility testing, and representativeness have been previously published [7]. In 2024, tetracycline minimum inhibitory concentration (MIC) values among the isolates ranged from 0.004 mg/L to > 256 mg/L, with MIC_50_ and MIC_90_ values of 1 mg/L (range in countries: 0.25–2 mg/L) and 32 mg/L (range in countries: 2–64 mg/L), respectively (Table 1). Based on the European Committee on Antimicrobial Susceptibility Testing (EUCAST) tetracycline resistance breakpoint (MIC > 0.5 mg/L) [8], 62.3% of isolates were resistant, with country-specific resistance levels ranging from 16.5% to 100% (Table 1).

**Table 1:** Tetracycline susceptibility and resistance in *Neisseria gonorrhoeae* isolates collected in 22 EU/EEA countries, 2024 (n = 3,579 isolates)

Countries (number of isolates)	MIC range mg/L	MIC_50_ mg/L	MIC_90_ mg/L	Resistant isolates EUCAST^a^		Resistant isolates CLSI^b^		Number of isolates with MIC > 2 mg/L		Number of isolates with MIC > 8 mg/L	
				Number	% or fraction^c^	Number	% or fraction^c^	Number	% or fraction^c^	Number	% or fraction^c^
Austria (n = 407)	0.125 – 128	1	32	307	75.4	174	42.8	97	23.8	85	20.9
Belgium (n = 184)	0.125 – 32	1	32	157	85.3	69	37.5	60	32.6	57	31.0
Bulgaria (n = 38)	1 – 64	1	64	38	38/38	19	19/38	9	9/38	8	8/38
Cyprus (n = 3)	0.25 – 2	0.25	2	1	1/3	1	1/3	0	0/3	0	0/3
Czechia (n = 106)	0.125 – 128	1	32	97	91.5	37	34.9	31	29.2	31	29.2
Denmark (n = 199)	0.016 – 32	0.5	8	45	22.6	37	18.6	36	18.1	17	8.5
France (n = 220)	0.064 – > 256	1	32	201	91.4	102	46.4	54	24.5	53	24.1
Germany (n = 200)	0.125 – 64	2	64	188	94.0	174	87.0	75	37.5	55	27.5
Greece (n = 100)	0.004 – 32	0.5	2	42	42.0	13	13.0	10	10.0	4	4.0
Hungary (n = 255)	0.064 – > 256	1	32	205	80.4	74	29.0	42	16.5	39	15.3
Iceland (n = 129)	0.25 – 64	1	32	111	86.0	51	39.5	33	25.6	32	24.8
Ireland (n = 243)	0.125 – 32	0.5	16	119	49.0	89	36.6	87	35.8	76	31.3
Italy (n = 100)	0.064 – 16	0.5	16	32	32.0	26	26.0	26	26.0	12	12.0
Luxembourg (n = 80)	0.032 – 32	0.5	32	40	50.0	19	23.8	17	21.3	17	21.3
Malta (n = 38)	0.125 – > 256	0.5	8	19	19/38	13	13/38	6	6/38	4	4/38
Norway (n = 394)	0.064 – 128	0.5	16	187	47.5	117	29.7	108	27.4	61	15.5
Poland (n = 18)	1 – 64	2	32	18	18/18	15	15/18	5	5/18	5	5/18
Portugal (n = 201)	0.5 – 128	2	64	196	97.5	184	91.5	74	36.8	67	33.3
Slovakia (n = 116)	0.125 – 64	1	16	68	58.6	35	30.2	27	23.3	24	20.7
Slovenia (n = 148)	0.064 – 64	0.5	16	31	20.9	18	12.2	17	11.5	16	10.8
Spain (n = 200)	0.032 – 32	0.25	8	33	16.5	31	15.5	30	15.0	3	1.5
Sweden (n = 200)	0.064 – 256	0.5	32	96	48.0	45	22.5	41	20.5	39	19.5
**Total (n = 3,579**)	**0.004** **– > 256**	**1**	**32**	**2,231**	**62.3**	**1,343**	**37.5**	**885**	**24.7**	**705**	**19.7**

CLSI: United States Clinical and Laboratory Standards Institute; EU/EEA: European Union/European Economic Area; EUCAST: the European Committee on Antimicrobial Susceptibility Testing; MIC: minimum inhibitory concentration.

^a^ Based on the clinical tetracycline resistance breakpoint (MIC > 0.5 mg/L) stated by EUCAST [8].

^b^ Based on the clinical tetracycline resistance breakpoint (MIC > 1.0 mg/L) stated by CLSI [10].

^c^ Percentages are presented unless the denominators are less than 60, in which case fractions are presented.

Among the 22 countries, 19 showed resistance rates > 30%, 13 resistance rates > 50%, and 10 resistance rates > 70%. These numbers are higher compared with the published Euro-GASP tetracycline resistance data from 2022 [9], when among a total of 19 countries submitting 4,787 isolates, 16 countries reported > 30% resistance, 11 > 50%, and seven > 70% (Table 1). In 2024, 37.5% (1,343/3,579) of isolates exhibited MICs > 1 mg/L, which corresponds to the United States (US) Clinical and Laboratory Standards Institute (CLSI) tetracycline resistance breakpoint [10]. Moreover 24.7% (885/3,579) had MICs > 2 mg/L, and 19.7% (705/3,579) had high-level resistance, i.e. MICs > 8 mg/L (Table 1), indicative of plasmid-mediated tetracycline resistance (www.clsi.org).

In 2023, 22 Euro-GASP countries provided 3,014 isolates for tetracycline-resistance analysis [11]. Compared with 2023, an increase in the proportion of tetracycline-resistant isolates with MICs of > 8 mg/L (p < 0.0002, z-test) occurred in 2024 and conversely, a decrease in the proportion of tetracycline-resistant isolates with MICs of 1–8 mg/L (p < 0.0017, z-test) (Figure).

**Figure:**
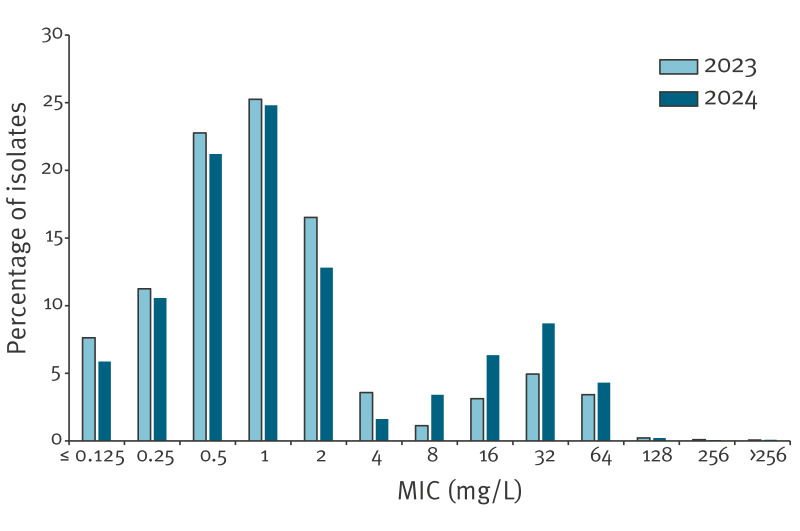
Tetracycline MIC distribution in *Neisseria gonorrhoeae* isolates collected in 22 EU/EEA countries in 2023^a^ and 2024^b^ (n = 3,014 and 3,579 isolates in each year)

EU/EEA: European Union/European Economic Area; MIC: minimum inhibitory concentration.

^a^ In 2023, the EU/EEA countries contributing isolates for tetracycline susceptibility testing were Austria, Belgium, Bulgaria, Czechia, Denmark, Estonia, France, Germany, Greece, Hungary, Iceland, Ireland, Italy, Malta, the Netherlands, Norway, Poland, Portugal, Slovakia, Slovenia, Spain, Sweden. Overall, in that year 58.4% of isolates were found resistant to tetracycline (i.e. MIC > 0.5 mg/L) [11].

^b^ In 2024, the EU/EEA countries contributing isolates for tetracycline susceptibility testing were Austria, Belgium, Bulgaria, Cyprus, Czechia, Denmark, France, Germany, Greece, Hungary, Iceland, Ireland, Italy, Luxembourg, Malta, Norway, Poland, Portugal, Slovakia, Slovenia, Spain, Sweden. Overall, in that year 62.3% of isolates were found resistant to tetracycline (i.e. MIC > 0.5 mg/L).

## Demographic and epidemiological correlations of tetracycline resistance, 2024

Associations between tetracycline resistance (MIC > 0.5 mg/L) and patient characteristics in 2024 were assessed using univariable and multivariable logistic regression. Odds ratios (ORs), 95% confidence intervals (CIs), and p values were calculated. Significant variables (p < 0.05) in the univariable analyses were included in a multivariable model adjusted for sex (male, female, other, unknown), age group, and mode of sexual transmission (categorised into MSM, men who have sex with women, or female individuals) (Table 2). Infection site was not included in the multivariable analyses as clinical sampling practice is not uniform across all countries. Only isolates from MSM remained independently associated with tetracycline resistance in the multivariable analyses (adjusted OR (aOR): 1.38; 95% CI: 1.06–1.79, p = 0.02) (Table 2).

**Table 2:** Assessment of the association between tetracycline resistance^a^ and patient characteristics by univariable and multivariable logistic regression analyses in 22 EU/EEA countries, 2024 (n = 3,579)

Characteristic	Proportion of isolates with tetracycline resistance % (95% CI)	Crude OR (95% CI)	Crude p value^b^	Adjusted OR (95% CI)	Adjusted p value^b^
**Sex (n = 3,448)**					
Female (n = 629)	53.6% (49.6–57.5)	Reference	< 0.001	Reference	0.164
Male (n = 2,819)	63.2% (61.4–65.0)	1.49 (1.25–1.77)		1.18 (0.93–1.51)	
**Age group (n = 3,443)**					
≤ 25 years (n = 1,040)	60.7% (57.6–63.7)	Reference	0.492	Reference	0.728
> 25 years (n = 2,403)	62.0% (60.0–63.9)	1.06 (0.91–1.23)		1.03 (0.86–1.23)	
**Sex and sexual transmission (n = 1,747)**					
Females (n = 629)	53.6% (49.6–57.5)	Reference	Reference	Reference	Reference
MSW (n = 395)	43.5% (38.6–48.6)	0.67 (0.52–0.86)	0.002	0.95 (0.70–1.26)	0.667
MSM (n = 723)	61.7% (58.0–65.2)	1.40 (1.12–1.73)	0.003	1.38 (1.06–1.79)	0.016
**Site of infection (n = 3,159)**					
Genital (n = 2,456)	65.2% (63.3–67.1)	Reference	Reference	NA	NA
Anorectal (n = 423)	63.1% (58.3–67.7)	0.91 (0.74–1.13)	0.409	NA	NA
Pharyngeal (n = 217)	47.0% (40.2–53.9)	0.47 (0.36–0.63)	< 0.001	NA	NA
Other (n = 63)	60.3% (47.2–72.4)	0.81 (0.49–1.35)	0.425	NA	NA

CI: confidence interval; EU/EEA: European Union/European Economic Area; EUCAST: the European Committee on Antimicrobial Susceptibility Testing; MIC: minimum inhibitory concentration; MSW: men who have sex with women only (heterosexual males); MSM: men who have sex with men; NA: not applicable (as clinical sampling practice is not uniform across all countries); OR: odds ratio.

^a^ Based on the clinical tetracycline resistance breakpoint (MIC > 0.5 mg/L) stated by EUCAST [8].

^b^ p value from logistic regression.

## Discussion

In our analysis, 62.3% of *N. gonorrhoeae* isolates from 22 EU/EEA countries in 2024 were resistant to tetracycline according to the EUCAST breakpoint (MIC > 0.5 mg/L) [8], and 37.5% had MIC values > 1 mg/L, corresponding to the US CLSI tetracycline resistance breakpoint [10]. For comparison, a recent global review (including 80,645 isolates across 51 countries, 1996–2023) [12], identified the highest tetracycline resistance levels in *N. gonorrhoeae* in the East Asia and Pacific (82.1%), as well as in the sub-Saharan African (81.6%) World Bank regions. Despite the global review finding lower proportions of tetracycline-resistant isolates in North America (26.5%), resistance levels there had increased nearly fourfold between 1996–2009 and 2010–2023 [12]. In line with the global review, a high tetracycline resistance level (average of 92% using EUCAST resistance breakpoint [8]) was found in eight countries participating in the World Health Organization Enhanced Gonococcal Antimicrobial Surveillance Programme (EGASP) in 2021–2024 [13], with all these countries located in the East Asia and Pacific and Sub-Saharan African regions.

The widespread tetracycline resistance in *N. gonorrhoeae* in the EU/EEA, will continue to undermine doxy-PEP’s effectiveness for gonorrhoea prevention in the EU/EEA. Among the isolates that we characterised in 2024, the percentages of tetracycline-resistant isolates with MICs > 8 mg/L rose compared to 2023, whereas those with MICs of 1–8 mg/L decreased [11]. Considering limitations arising from minor variations in countries participating in Euro-GASP each year, this finding should be interpreted with caution. However, it may suggest a transition from low-level chromosomal tetracycline resistance (MIC: 1–8 mg/L) towards high-level plasmid-*tetM*-mediated tetracycline resistance (MIC > 8 mg/L) and a reduction in tetracycline susceptible gonococcal isolates, both of which may indicate a shift in circulating gonococcal strains and/or increasing tetracycline selective pressure.

In this regard, recent data suggest that doxy-PEP may drive tetracycline resistance selection in *N. gonorrhoeae*. In King County, Washington, US, the tetracycline resistance level in *N. gonorrhoeae* among MSM was stable (ca 27%) from 2017 to the first quarter (Q1) of 2023 but increased to 70% by Q2 of 2024 (p < 0.0001), coinciding with local doxy-PEP guidelines being released in Q2 2023 [14]. High-level tetracycline resistance increased from 2% to 65% between 2021 and 2024 (p < 0.0001). Frequent doxy-PEP use (≥ 3 doses/month) was significantly associated with both overall resistance and high-level resistance to tetracycline (p ≤ 0.01), whereas 1–2 doses were not associated [14]. In the DOXYVAC study (2021–2022), doxy-PEP was associated with an increase in *N. gonorrhoeae* isolates with high-level resistance to tetracycline plus decreased susceptibility to cefixime [2,15].

Notably, the association found in the current work between tetracycline resistance and isolates from MSM populations, the key target population for doxy-PEP particularly for syphilis control, re-enforces previous studies that show doxy-PEP will have limited impact on gonorrhoea incidence due to tetracycline resistance, and may further increase resistance due to additional selective pressure and further development of high-level tetracycline resistance through prophylactic use of doxycycline.

The impact of doxy-PEP may also extend to bystander organisms and microbiomes, including their resistomes. Limited data for *Staphylococcus aureus* [14,16], *Streptococcus pyogenes* [14] and microbiomes [17,18] have been published and discussed elsewhere [19,20].

The main limitations of Euro-GASP include that not all EU/EEA countries culture gonococcal isolates and can participate, some additional countries do not participate each year, the number of isolates in some participating countries is low, and the coverage in the reporting of some patient characteristics, e.g. sexual transmission, is suboptimal. Nevertheless, Euro-GASP continuously works to overcome these limitations and overall, Euro-GASP data have been shown to adequately reflect the EU/EEA antimicrobial resistance situation [7].

## Conclusions

Adoption of doxy-PEP in European settings should be cautious and targeted, with consideration of the risks of selecting doxycycline and tetracycline resistance in both target pathogens and bystander organisms. It is not suitable for gonorrhoea prevention, and if implemented for syphilis or chlamydia, doxy-PEP should be integrated as one component of a broader, comprehensive STI prevention strategy that includes routine testing, rapid linkage to care, partner notification, condom access, relevant vaccinations, behavioural interventions, antimicrobial stewardship and optimised treatment of STIs in accordance with current evidence-based European guidelines.

## Data Availability

The first author (SJ) and the last author (MU) had full access to all the data in the study and datasets can be made available from the corresponding author after publication on reasonable request.

## Euro-GASP tetracycline study group members

Austria: Sonja Pleininger, Stefanie Schindler, Ziad El-Khatib. Belgium: Irith De Baetselier, Dominique Van Beckhoven, Dorien Van den Bossche, Amaryl Lecompte. Bulgaria: Ivva Philipova. Cyprus: Panayiota Maikanti Charalambous, Despo Pieridou, Olga Apostolou. Czechia: Hana Zákoucká, Helena Žemličková, Vladislav Jakubů. Denmark: Steen Hoffmann, Maria Wessman. France: Beatrice Bercot, Emilie Chazelle, Cheick Kounta, Gilles Delmas. Germany: Dagmar Heuer, Regina Selb, Klaus Jansen. Greece: Vivi Miriagou, Eirini Siatravani, Dimitra Paraskeva. Hungary: Eszter Balla. Iceland: Lena Rós Ásmundsdóttir, Anna Margret Gudmundsdottir, Marianna Thordardottir, Kristjan Orri Helgason. Ireland: Brendan Crowley, Sinead Saab, Derval Igoe, Mark Campbell, Angeline McIntyre. Italy: Paola Stefanelli, Barbara Suligoi. Luxembourg: Monique Perrin. Malta: Francesca Mifsud, Julie Haider, Robert Cassar. Norway: Bente Børud, Hilde Kløvstad. Poland: Beata Młynarczyk-Bonikowska. Portugal: Maria José Borrego. Slovakia: Peter Pavlik, Alexandra Bražinová. Slovenia: Julija Germ, Tanja Kustec. Spain: Raquel Abad, Julio Vazquez Moreno, Javier Gómez Castellá. Sweden: Magnus Unemo, Susanne Jacobsson, Daniel Schröder.
